# Effect of Gum Arabic (*Acacia Senegal*) supplementation on visceral adiposity index (VAI) and blood pressure in patients with type 2 diabetes mellitus as indicators of cardiovascular disease (CVD): a randomized and placebo-controlled clinical trial

**DOI:** 10.1186/s12944-018-0711-y

**Published:** 2018-03-20

**Authors:** Rasha Babiker, Khalifa Elmusharaf, Michael B. Keogh, Amal M. Saeed

**Affiliations:** 1 Department of Physiology, Faculty of Medicine, University of Medical Sciences & Technology, Khartoum, Sudan; 2 Graduate Entry Medical School, University of Limerick, Limerick, Ireland; 3 Human Biology & Research Laboratory Manager, School of Medicine, Royal College of Surgeons in Ireland RCSI-Bahrain, P.O Box 15503, Adliya, Manama, Bahrain; 4 Department of Physiology, Faculty of Medicine, University of Khartoum, P.O Box 102, Khartoum, Sudan

**Keywords:** Type 2 diabetes mellitus, Gum Arabic, VAI, Lipid profile, Nutrition, BMI and blood pressure

## Abstract

**Background:**

There is a strong association between cardiometabolic risk and adipose tissue dysfunction with great consequences on type 2 diabetic patients. Visceral Adiposity Index (VAI) is an indirect clinical marker of adipose tissue dysfunction. Gum Arabic (GA) is a safe dietary fiber, an exudate of *Acacia Senegal.* Gum Arabic had shown lipid lowering effect in both humans and animals. The aim of this trial was to determine the effect of GA supplementation on anthropometric obesity marker, Visceral Adiposity Index (VAI) and blood pressure in patients with type 2 diabetes mellitus.

**Methods:**

This randomized, double blinded, placebo controlled trial recruited a total of 91 type 2 diabetic patients (73 females, 18 males), age (mean ± SD) 50.09 ± 9.3 years on hypoglycemic agents and were randomly assigned into two groups, either to consume 30 g of GA or 5 g of placebo daily for 3 months. Anthropometric obesity markers were measured and indices were calculated. Blood pressure was measured and high density lipoprotein (HDL) and triglycerides (TG) were determined in fasting blood samples at the start and end of the study period.

**Results:**

After intervention, Gum Arabic decreased BMI and VAI significantly (*P* < 0.05) in GA group by 2 and 23.7% respectively. Body adiposity index significantly decreased by 3.9% in GA group while there were no significant changes in waist circumference or waist-to-hip ratio (WHR). Systolic blood pressure significantly decreased by 7.6% in GA group and by 2.7% in placebo group from baseline with no significant changes in diastolic blood pressure in the two groups.

**Conclusion:**

Gum Arabic consumption at a dose of 30 g/d for 3 months may play an effective role in preventing weight gain and modulating adipose tissue dysfunction in type 2 diabetic patients, although no effect has been shown in waist-to-hip ratio.

**Trial registration:**

The trial had been registered as prospective interventional clinical trials in the Pan African Clinical Trial Registry (PACTR) PACTR201403000785219, on 7th March 2014.

## Background

The incidence of diabetes is increasing globally leading to serious complications [1]. In Sudan, knowledge about and awareness of diabetes is poor and that adversely affects its control and management [2].

The use of oral hypoglycemic drugs is associated with the tendency to gain weight or other side effects; therefore nutritional care is crucial for diabetic patients.

Obesity and weight gain play a major role in insulin resistance and development of diabetes. It was recognized that elevated levels of triglycerides lead to elevated levels of free fatty acids which modulate pathways that link insulin receptors with glucose transporters and impair normal function of beta cell. Furthermore, hyperlipidaemia can lead to more difficulties in controlling hyperglycaemia in diabetic patients [3]. The presence of low density lipoprotein (LDL) particles and low HDL levels are among the components of cardiometabolic syndrome and its risk can be identified by elevated TG/HDL ratio [4].

In clinical practice, anthropometric measurements such as body mass index could be a predictor for the management of overweight or obesity in patients at high cardiometabolic risk. Unfortunately, there is conflicting evidence in predicting CVD in diabetic patients due to variation in visceral adipose tissue accumulation by many etiological factors including age, gender and ethnicity and differences in body fat distribution and composition [5], but generally there is a linear relationship between BMI and cardiovascular disease.

Visceral adipose index is a good predictor of visceral adiposity which is related to type 2 diabetes [6, 7] and hypertension [8]. VAI is indicative of fat distribution and an indicator of altered adipose function which is associated with insulin resistance, an increased VAI is associated with an increased risk of abdominal obesity, hypertriglyceridemia and low HDL [9], and it has been proven to be superior to other adiposity indices in predicting CVD risk [10] .

In 2011, the Body Adiposity Index (BAI) was proposed to provide a direct estimate of percentage of body adiposity [11]. Other simple anthropometric measurements had also shown strong association with cardiometabolic risk [12].

Diet with high fiber prevents visceral adiposity reflected by reduction in waist circumference and BMI [13].

Gum Arabic (GA) with its high fiber content has been reported to improve lipid metabolism in experimental animal studies with antioxidant effect and improving capillary function. Consequently we postulated that consumption of GA would lead to improvement of anthropometric indices and blood pressure as indicators of CVD. Therefore, this study was designed to determine effects of daily intake of 30 g Gum Arabic on anthropometric variables associated with cardiometabolic risk among type 2 diabetes.

## Methods

### Study design

This was a single center, randomized, double blind, placebo-controlled trial comparing pre and post effects of GA versus placebo in diabetic patients who have agreed to participate in this trial.

The trial was conducted at Academy Charity Teaching Hospital (ACTH) in Sudan; it took place from August 2014 to February 2015. All patients had been diagnosed with type 2 diabetes mellitus according to the criteria of WHO [14].

The trial protocol was approved by the local ethical committee of State of Khartoum Ministry of Health –and it is registered under PACTR201403000785219. This study was conducted according to the guidelines of the Declaration of Helsinki. Informed consent was obtained from all patients.

### Outcomes

#### Primary outcome

The primary outcome was assessed in our previous article which was the effect of GA on mean percent change of fasting plasma glucose and HbA1c from baseline level [15].

#### Secondary outcome

Secondary outcomes included in our previous article were changes of GA on BMI and lipid profile [15]. In this study secondary outcomes included are obesity anthropometric indexes (VAI, BAI, LAP and deep abdominal adipose tissue (DAAT) and mean percent change of systolic and diastolic blood pressure from baseline level.

### Recruitment

**Fig. 1:**
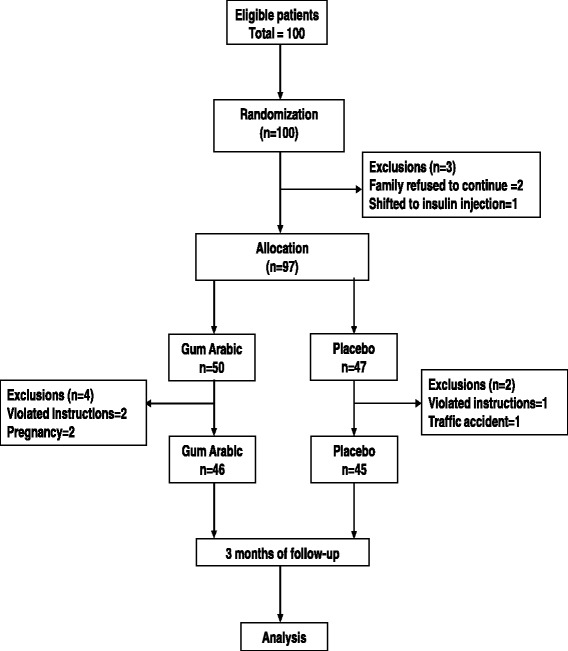
flowchart of study design

Patients were recruited from diabetes clinic at ACTH, Khartoum (Fig. 1). Those referred to the clinic were offered the opportunity to participate in this trial. The initial screening visit included the signing of the consent form, filling of questionnaire, and measurement of weight, height, waist circumference, hip circumference, blood pressure and blood sample for biochemical test. Patients were instructed to fast for 10–12 h prior to their first visit.

A total of 100 type 2 diabetic patients agreed to take part and signed the consent forms after the purpose and instructions concerning the trial had been explained to them. Patients with type 2 diabetes mellitus and on anti-hyperglycemic medication with fasting plasma glucose of (FPG) ≥7.0 mmol/L (126 mg/dl) and HbA1c ≥ 6.5% were included.

Alcoholics or drug addicts, patients with history of gastrointestinal diseases or Gum Arabic allergy, type 1 diabetes mellitus, pregnancy or planning to have a child within next 6 months and patients not eligible for the study due to medical reason evaluated by physician were excluded [15].

### Randomization and blinding

Randomization was done by series of numbers generated by independent third-party not associated with the study. Patients and chief attending doctor were blind to the intervention. Supplement package of GA or placebo (pectin) were prepared in sealed boxes. Pectin was chosen as placebo because it is a soluble polysaccharide with viscous sensation when dissolved in water similar to GA. It is a good source of fiber and has been recommended for diabetics.

The daily supplement was a 30 g of powdered GA (Dar Savanna Ltd., Khartoum, Sudan) or 5 g of placebo (Andre Pectin, Yantani, China). The high viscosity of pectin limits consumption of it to 5 g only, 250 mL of water was mixed well with the package content before intake. No dietary restrictions were given and patients were asked not to change their lifestyle or physical activities during the study period. Patients continued taking their medications as prescribed by the physician.

Consumption of supplements and any of adverse reactions were examined by attending doctor. Lifestyle and medication of each patient was checked using a self-reporting sheet. Participants were followed weekly and the final examination was completed after 3 months of intervention [15].

### Intervention

Of the 100 patients who were enrolled in the research, 97 were given either placebo (*n* = 47) or GA (*n* = 50). Ninety-one completed the study, three patients violated the instructions and were advised to discontinue; two got pregnant and one patient had a traffic accident and was hospitalized.

### Data collection and measurements

Baseline measurements for all patients were taken, and they included; anthropometric, blood pressure measurements, blood samples for fasting plasma glucose (FPG), HbA1c and lipid profiles, in addition to completing a general health questionnaire. Blood samples were obtained by venipuncture kept in cryogenic collection tubes and were centrifuged immediately at 2700 rpm for 10 min to separate serum from blood. The serum was used to determine lipid profile (HDL and triglyceride levels). Enzymatic biochemical analyses were performed using (BioSystems S.A. 310- Semi automated chemistry analyser) to determine lipid profile; HDL and LDL were analyzed by using precipitation and cholesterol oxidase method, FPG was analysed by using glucose oxidase method and HbA1c by direct ion exchange method [15]. Waist circumference was measured by using flexible, elastic measuring tape at midpoint between the lower rib and the top of iliac crest at the end of expiration. Hip circumference was measured at the level of the greater femoral trochanters. The waist circumference divided by the hip circumference gave the (waist-to-hip ratio (WHR). Body weight (kg) was measured on calibrated balance scale and height (cm) was measured with standmeter to nearest 0.5 cm, BMI was calculated as the ratio of weight in kilograms to height in meters squared (kg/m^2^) [16].

Lipid accumulation product **(**LAP**)** was calculated with the following formula: male LAP = [waist (cm)-65] X TG (mmol/l) and female LAP = [waist (cm) -58] X TG (mmol/l) [17], and VAI was calculated from [WC/ (39.68 + 1.88 x BMI)] x (TG/1.03) x (1.31/HDL) for men and [WC/ (36.58 + 1.89 x BMI] x (TG/0.81) x (1.52/HDL) for women [18].

Body Adiposity Index (BAI) was calculated by using hip circumference and height (BAI = hip circumference (cm) divided by (height (m)) ^1.5^ minus 18) [11].

Deep abdominal adipose tissue (DAAT) was calculated by using the formula: − 382.9 + [1.09 x weight - (kg)] + [6.04 x WC- (cm)] + (− 2.29 x BMI) for men and − 278 + [− 0.86 x weight - (kg)] + [5.19 x WC- (cm)] for women [16].

Blood pressure (BP) was measured using mercury sphygmomanometer.

Visceral adipose index (VAI) and blood pressure (BP) were measured as primary outcome measures, HC, WC, WHR, BMI, BAI, LAP, DAAT, HDL-c and triglyceride were evaluated and measured as secondary outcomes. These measurements were performed before and after intervention.

### Data analysis

Statistical analyses were performed using SPSS (Statistical Package for the Social Science, IBM SPSS version 23) values were expressed as mean ± standard deviation and Paired sample T-Test was used to compare continuous variables or Pearson’s chi-square test within the groups. *P* < 0.05 was regarded as statistically significant.

## Results

### General characteristics of participants

A total of 120 patients were invited with 100 patients identified as eligible to participate in this study, nine patients were excluded for not meeting the project criteria. Ultimately, a total of 97 patients were randomly assigned to Gum Arabic or placebo group as shown in study flowchart in Fig. 1.

### Demographic data and baseline parameters

Demographic characteristics of participants and baseline parameters are reported in Table 1. No significant difference in patient’s baseline characteristics between the two groups (*P* > 0.05), however, the majority of patients were females (80.2%).

**Table 1:** Baseline characteristic and risk factors of patients in Gum Arabic and Placebo groups (*n* = 91)

Variables	Gum Arabic (*n* = 46)		Placebo (*n* = 45)				
		SEM		SEM	*P* value	95 % C.I of differences	
						Lower Upper	
Age (Years) ^a^	49.96 ± 8.73	1.29	50.22 ± 9.29	1.39	0.89	−4.02	3.49
Male ^b^	12(26.1%)		6(26.1%)		0.19		
Female ^b^	34(73.9%)		39(86.7%)				
Duration of DM (months) ^a^	64.04 ± 55.43	8.17	59.53 ± 48.09	7.17	0.68	−17.13	26.15
Height (cm) ^a^	162.26 ± 9.01	1.33	161.07 ± 8.82	1.31	0.52	−2.52	4.91
Weight (kg) ^a^	72.86 ± 15.72	2.32	77.07 ± 13.84	2.06	0.18	−10.38	1.96
Waist circumferences (cm) ^a^	98.65 ± 12.41	1.83	94.76 ± 14.9	2.22	0.18	−1.81	9.60
WHR ^a^	0.93 ± 0.08	0.011	0.89 ± 0.10	0.015	0.08	−0.00	0.067
BMI (Kg/m^2^) ^a^	27.66 ± 5.37	0.79	29.96 ± 6.54	0.97	0.07	−4.79	0.19
BAI ^a^	33.90 ± 7.46	1.10	34.38 ± 8.65	1.29	0.77	−3.64	2.72
VAI ^a^	3.17 ± 2.29	0.34	2.08 ± 1.55	0.23	0.01	0.24	2.00
DAAT,cm^2 a^	190.92 ± 70.88	10.45	153.65 ± 67.37	10.04	0.01	5.61	62.90
LAP,cm.mmol/l ^a^	61.28 ± 39.46	5.81	41.65 ± 32.86	4.90	0.01	2.66	36.58
SBP (mmHg) ^a^	131.74 ± 16.37	2.43	128.44 ± 14.45	2.19	0.312	−3.14	9.73
DBP (mmHg) ^a^	80.00 ± 9.89	1.42	78.22 ± 8.87	1.35	0.148	−2.14	5.69
TG (mmol/l) ^a^	1.54 ± 0.63	0.093	1.10 ± 0.54	0.08	0.00	0.156	0.72
HDL(mmol/l) ^a^	1.10 ± 0.39	0.058	1.15 ± 0.39	0.059	0.57	−0.22	0.12
Hypertension ^b^	14(30.4%)		6(13.3%)		0.07		
Smoking ^b^	5(10.9%)		2(4.4%)		0.43		
Exercise this year ^b,c^	3(6.5%)		2(4.4%)		0.66		
Alcohol consumption ^b^	2(4.3%)		2(4.4%)		0.98		

Values are presented as ^a^ Mean ± SD, ^b^ number (percentage) [15]

Abbreviations: *BMI* Body Mass Index, *SBP* Systolic Blood Pressure *DBP* Diastolic Blood Pressure

^c^ Exercise in year 2014 to 2015

In Table 1, cardiometabolic risk parameters including blood pressure, smoking, physical activity and alcohol consumption did not differ significantly by group. About 59.4% (*n* = 54) of patients were on combined therapy (sulfonylureas e.g. (glimepiride and glipizide) and metformin) and 40.6% (*n* = 37) on metformin only [15].

### Changes in biochemical parameters

High density lipoprotein had significantly increased by 20% from baseline in GA group with no significant change in placebo group. Triglycerides had not statistically changed in both groups (Table 2).

**Table 2:** Anthropometric and biochemical in study groups pre and post intervention

	Gum Arabic (*n* = 46)						Pectin (*n* = 45)					
	Pre	Post	Mean difference	95 % CI of the Difference		P value	Pre	Post	Mean difference	95 % CI of the Difference		*P* value
				Lower	Upper					Lower	Upper	
Weight (kg)	72.86 ± 15.72	71.40 ± 16.26	1.45	0.42	2.49	0.007	77.07 ± 13.84	77.08 ± 13.83	−0.01	−0.05	0.02	0.437
Waist C.(cm)	98.65 ± 12.41	97.35 ± 11.98	1.30	−0.36	2.97	0.121	94.76 ± 14.90	96.13 ± 15.18	−1.38	−3.32	0.56	0.159
Hip C.(cm)	106.76 ± 13.54	104.07 ± 12.38	2.70	0.77	4.62	0.007	106.44 ± 14.67	103.84 ± 14.04	2.60	−0.94	6.14	0.146
TG (mmol/l)	1.54 ± 0.63	1.37 ± 0.59	0.17	−0.01	0.35	0.061	1.10 ± 0.54	1.10 ± 0.53	0.00	−0.03	0.03	0.958
HDL(mmol/l)	1.10 ± 0.39	1.32 ± 0.61	−0.22	− 0.38	−0.05	0.011	1.15 ± 0.39	1.16 ± 0.40	−0.01	−0.04	0.02	0.442
WHR	0.93 ± 0.08	0.94 ± 0.08	−0.01	−0.03	0.01	0.258	0.89 ± 0.10	0.92 ± 0.09	−0.02	−0.05	0.01	0.139
BMI (kg/m^2)^	27.66 ± 5.37	27.09 ± 5.49	0.57	0.16	0.98	0.007	29.96 ± 6.54	29.96 ± 6.54	0.00	−0.02	0.01	0.501
BAI	33.90 ± 7.46	32.58 ± 6.85	1.32	0.36	2.27	0.008	34.38 ± 8.65	33.10 ± 8.21	1.29	−0.49	3.07	0.152
VAI	3.17 ± 2.29	2.42 ± 1.82	0.75	0.11	1.38	0.022	2.08 ± 1.55	2.05 ± 1.48	0.03	−0.06	0.12	0.469
DAAT,cm^2^	190.92 ± 70.88	184.85 ± 66.48	6.07	−2.49	14.62	0.16	153.65 ± 67.37	160.86 ± 69.39	−7.21	−17.51	3.08	0.165
LAP,cm.mmol/l	61.28 ± 39.46	52.44 ± 35.29	8.84	1.18	16.49	0.025	41.65 ± 32.86	42.77 ± 30.23	−1.12	−3.61	1.36	0.367
SBP (mmHg)	131.74 ± 16.37	121.74 ± 8.77	10.00	6.69	13.31	0.000	128.44 ± 14.45	125.11 ± 12.90	3.33	0.77	5.90	0.012
DBP (mmHg)	80.00 ± 9.89	77.61 ± 8.48	2.39	−0.49	5.27	0.102	78.22 ± 8.86	78.44 ± 8.52	−0.22	−2.82	2.38	0.864

Values are presented as (Mean ± SD); Abbreviations: *Hip C* Hip circumference, *TG* Triglycerides, *HDL* High density lipoprotein, *WHR* Waist to hip ratio, *BMI* Body Mass Index, *BAI* Body Adiposity Index, *VAI* Visceral Adiposity Index, *DAAT* Deep abdominal adipose tissue**,** *LAP* Lipid accumulation product, *SBP* Systolic Blood Pressure and DBP: Diastolic Blood Pressure

### Changes in anthropometric and indexes

Gum Arabic consumption significantly decreased weight and hip circumferences by 2% and 2.5% respectively from baseline level, although no significant change in waist circumference was observed among this group (*P* = 0.121).

BMI had significantly decreased by 2% in GA treated group from baseline level (*P* = 0.007).

Visceral adiposity index and BAI were reduced by 23.7% and 3.9% respectively among GA group from baseline level. Lipid accumulation product **(**LAP**)** significantly reduced in GA group by 14.4% from baseline level but no significant change was observed for deep abdominal adipose tissue (DAAT).

### Changes in blood pressure

Twenty nine point nine percent of total patients were hypertensive on hypertensive medications mainly (beta blockers, calcium channel blockers or angiotensin converting enzyme inhibitors) beside antidiabetic medication. Systolic blood pressure was significantly reduced in both groups with no significant effect on diastolic blood pressure in both groups as seen in Table 2.

## Discussion

Cardiovascular diseases incidence is increased in people with type 2 diabetes [19]. Prevention and treatment of diabetes play a beneficial role in the delay of complications and improving quality of life in diabetic patients. Gum Arabic is a soluble fermentable fiber has shown hypoglycaemic, antioxidant effects and also improved lipid metabolism in previous studies [20].

Gum Arabic showed anti-obesity effect among healthy adults [21], and blunted weight gain in experimental studies in animals [22]. According to our knowledge, this is the first trial studying the effect of GA on simple anthropometric measurements and approximate visceral adiposity by measuring VAI and LAP among type 2 diabetic patients. Our data showed that supplementation of GA significantly decreased BMI among GA group with no effect on WC. The negative effect of GA on WC might be explained by the blotted abdomen caused by fermentation of GA in colon and gases formation which might affect WC measurement. However, BMI and WC reflects only excess weight with poor sensitivity in diagnosing excess body fatness and differentiation between subcutaneous and visceral fat compartments [23]. Therefore, an increased WC cannot always reflect high-risk visceral fat [24]. Individuals with different risk levels of CVD and diabetes may have similar BMI, while their WC and metabolic risk profiles may be different. Visceral fat is characterized by different levels hence there is different levels of insulin resistance [25]. It is also linked to several pathological conditions including impaired glucose and lipid metabolism and insulin resistance [26, 27]. Visceral fat also determines cardiovascular risk profile and increases the susceptibility of arterial hypertension [28]. Previous study by Glover et, al (2009) reported that intake of dietary fiber, including GA was associated with a significant fall in mean systolic blood pressure [SBP] in normal individuals who neither had hypertension nor diabetic [29]. In the current study there was significant reduction of [SBP] by 7.6% from baseline level within GA group vs. 2.7% reduction in placebo group and there was insignificant reduction of [DBP] in both groups. In this work about 30% of GA patients were hypertensive compared to 13% in placebo group as a result of the randomization; this created a difficulty in assessing the effect of GA on the mean blood pressure in this study. Lipid accumulation product **(**LAP**)** is associated with diabetes, predicting CVD and metabolic diseases better than WC and BMI [17]. Our data showed significant decrease of VAI and LAP in GA group, such VAI and LAP are calculated with WC, BMI, triglyceride and HDL. In this study there was a decrease in blood triglyceride by 11% which may have a positive effect on a glucose metabolism, as high triglyceride interferes with insulin level and actions [30]. The obtained reduction in VAI and the significant increase in HDL level can possibly improve glucose homeostasis by different mechanisms and enhance insulin sensitivity [31]. Gum Arabic itself is reported to reduce TNFa expression in visceral adipose tissues of female mice and its production is linked to obesity-insulin resistance [32].

The dyslipidaemia in patients with type 2 diabetes (high triglyceride and low HDL-C levels) presents a major risk of cardiovascular disease. Since this was decreased by GA intake in addition to the improvement in body mass composition we can claim that GA can be a beneficial supplementation in diabetic type 2 patients [30].

## Conclusions

The findings of this study demonstrated that GA consumption in a dose of 30 g /day was effective in weight reduction and modulating adipose tissue dysfunction in type 2 diabetic patients. This was reflected by the increase in HDL and decrease of triglyceride which were major component in assessing the CV risk factors in diabetic patients.

## Abbreviations

11β-HSD1: 11β-hydroxysteroid dehydrogenase type I
BAI: Body adiposity index
BMI: Body mass index
BP: Blood pressure
DAAT: Deep abdominal adipose tissue
GA: Gum Arabic
HDL: High-density lipoprotein
LAP: Lipid accumulation product
LDL: Low-density lipoprotein
TG: Triglycerides
TNFa: Tumor necrosis factor alpha
VAI: Visceral adiposity index
WC: Waist circumference
WHR: Waist to hip ratio
